# Real-Time Production of PGA, PGV, Intensity, and Sa Shakemaps Using Dense MEMS-Based Sensors in Taiwan

**DOI:** 10.3390/s21030943

**Published:** 2021-01-31

**Authors:** Benjamin M. Yang, Himanshu Mittal, Yih-Min Wu

**Affiliations:** 1 Department of Geosciences, National Taiwan University, Taipei 10617, Taiwan; f06224222@ntu.edu.tw (B.M.Y.); hmittal@jpr.amity.edu (H.M.); 2 Centre for Ocean-Atmospheric Science & Technology (COAST), Amity University Jaipur, Rajasthan 303002, India; 3 Institute of Earth Sciences, Academia Sinica, Taipei 11529, Taiwan; 4 Research Center for Future Earth, National Taiwan University, Taipei 10617, Taiwan

**Keywords:** MEMS accelerometers, real-time shakemaps, earthquake early warning, seismic hazard mitigation, *P*-Alert

## Abstract

Using low-cost sensors to build a seismic network for earthquake early warning (EEW) and to generate shakemaps is a cost-effective way in the field of seismology. National Taiwan University (NTU) network employing 748 P-Alert sensors based on micro-electro-mechanical systems (MEMS) technology is operational for almost the last 10 years. This instrumentation is capable of recording the strong ground motions of up to ± 2g and is dense enough to record the near-field ground motion. It has proven effective in generating EEW warnings and delivering real-time shakemaps to the concerned disaster relief agencies to mitigate the earthquake-affected regions. Before 2020, this instrumentation was used to plot peak ground acceleration (PGA) shakemaps only; however, recently it has been upgraded to generate the peak ground velocity (PGV), Central Weather Bureau (CWB) Intensity scale, and spectral acceleration (*S_a_*) shakemaps at different periods as value-added products. After upgradation, the performance of the network was observed using the latest recorded earthquakes in the country. The experimental results in the present work demonstrate that the new parameters shakemaps added in the current work provide promising outputs, and are comparable with the shakemaps given by the official agency CWB. These shakemaps are helpful to delineate the earthquake-hit regions which in turn is required to assist the needy well in time to mitigate the seismic risk.

## 1. Introduction

**Figure 1:**
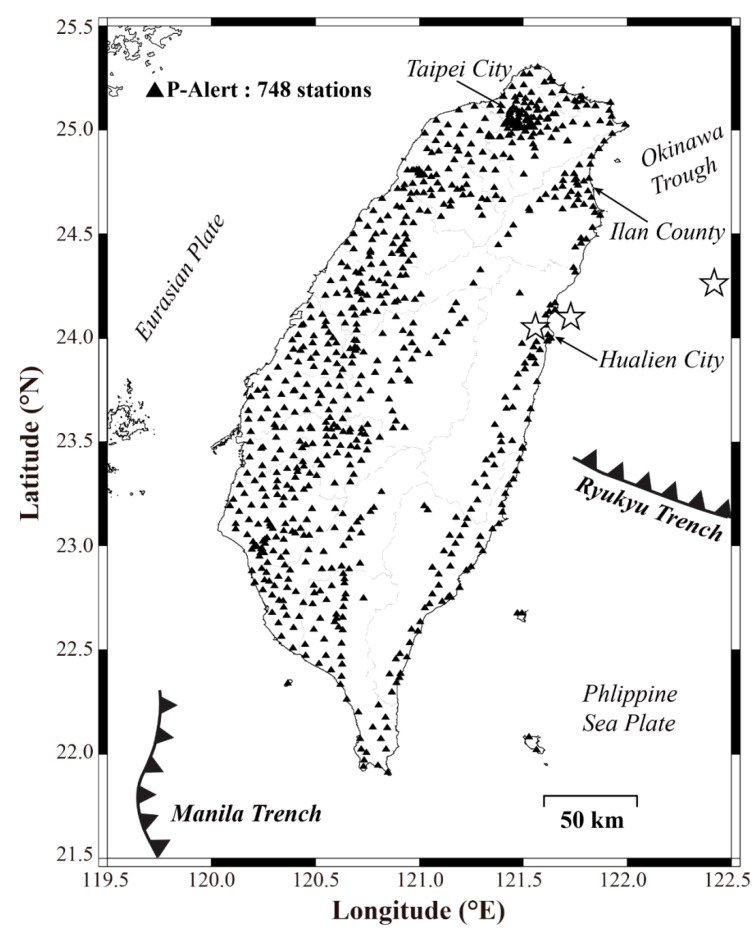
The seismotectonic settings of Taiwan along with the distribution of P-Alert instruments in different parts. The stars represent the epicenters of the earthquakes mentioned in Section 4 and Section 6.

Taiwan, having an approximate area of 36,193 km^2^, has a complex tectonic structure and sits at the junction of the Luzon Island arc and the Ryukyu Island Arc. In Eastern Taiwan, the Philippine Sea plate subducts under the Eurasian plate along the Ryukyu Trench (Figure 1), and off the southern tip of Taiwan, the Eurasian plate subducts under the Philippine Sea plate [1]. Because of this on-going activity, the country is repeatedly rocked by high and intermediate magnitude earthquakes. Many faults (thrust and strike-slip) responsible for these earthquakes are identified all over the country. Most of the earthquakes occur in Taiwan as a result of arc-continent collision and subduction, and sometimes the higher magnitude earthquakes occurring along this boundary pose threat to life and property. As earthquakes are unpredictable, therefore, some other measures of protecting human lives and property damage should be explored.

The EEW system is one of the best ways developed in previous decades and is operational in many areas of the world based on regional and onsite algorithms. The concept of regional EEW lies in detecting ground movement in the early stage of an earthquake and transmitting the ground motion data recorded by the instruments to the central recording system for computation of various parameters, which can be used to issue the warning during an earthquake. A regional EEW system consisting of several instruments installed within a specified area is used to record the earthquake. The onsite EEW system, which determines the earthquake parameters from the initial portion of the P waves and predicts the more severe ground shakings of the following S-wave trains. The CWB is the official agency for reporting EEW in Taiwan [2]. The EEW system of CWB uses around 140 instruments installed in different parts of the country [3]. Currently, the CWB network acts as a regional EEW system and issues the warning to the cities around 50 km away from the epicenter. For all the cities lying within 50 km, it is a blind zone and no warning is possible under this system. In addition to this official EEW network established by CWB, the other two EEW networks established by the National Center for Research on Earthquake Engineering (NCREE), and National Taiwan University (NTU) are also in operation. The NCREE network is an on-site and regional hybrid network and uses around 90 instruments installed in various elementary schools [4]. The CWB and NCREE networks work perfectly but sometimes the denser networks are required to perform additional tasks, for example, to plot the detailed shakemaps for the rapid post-earthquake response. As both CWB, as well as NCREE networks, use traditional high-cost seismographs or accelerographs, installing more instruments will be an expensive process.

The EEW indeed requires densely spaced instruments to perform in a better way, therefore, various countries including Taiwan are working on developing substitute instruments that may be affordable than the traditional ones. The MEMS accelerometers for seismological studies and especially EEW were introduced in the 1990s [5]. These sensors are tiny, cost-effective, and ideal for recording near-source high-frequency ground motion. The applicability and usefulness of these MEMS-based instruments in different applications including EEW and recording aftershock activity are reported by various researchers [6,7,8,9,10,11,12,13,14,15]. With the advancement in MEMS-based technology, many countries and companies have developed their sensors and are using them widely for EEW [16,17,18,19].

The NTU network uses the low-cost accelerographs known as *P*-Alert for the configuration of the EEW system. These *P*-Alert sensors use MEMS accelerometers housed on a small printed circuit board. These sensors are developed by the research team at NTU in collaboration with a technology company. These newly introduced sensors have reduced the cost to around 1/10 times of the traditional instruments. Like most of the MEMS-based networks around the world, *P*-Alert sensors with low dynamic range and low sensitivity belong to Class-C type [9]; therefore, the NTU network tries to focus on the amplitude parameters instead of those parameters which work stably only with high signal-to-noise ratio (SNR) data, for example, *P*-wave arrival time or τc. To maximize the advantage of MEMS sensors, the NTU network has dedicated to generating intensity shakemaps which are indeed helpful for hazard mitigation and relief for the past few years. Individual P-Alert devices can not only provide continuous earthquake waveform to the processing center but also give onsite early warning based on local *P*-waves shaking that makes these devices popular to the public. And the low-cost of *P*-Alert instruments allow building a denser network in different parts of the country. Currently, around 748 instruments with an average spacing of around 5 km are installed.

## 2. The NTU Network

The NTU network consists of first-generation *P*-Alert and second-generation *P*-Alert plus instruments. The *P*-Alert plus instrument is an improved version of *P*-Alert, obtained by overcoming the drawbacks of *P*-Alert, especially related to dynamic range and storage. *P*-Alert sensors have 16-bit resolution and had a problem in picking the smaller earthquakes, but this drawback has been improved in *P*-Alert plus that uses 24-bit tri-axial MEMS.

The first purpose of the NTU network is to record the earthquakes, and process these records over different frequency ranges to calculate various parameters in real-time [20]. Regularly, various parameters essential for EEW are calculated, and once the calculated parameters hint at the earthquake magnitude ≥5.5, the warning is disseminated to disaster relief agencies to take proper action. Other than EEW, NTU network data is also used for other applications including shakemaps plotting and seismological studies. The data recorded by this network is also archived for future use for seismology and engineering researches such as post-earthquake structure health evaluation [21]. The successful functioning of this *P*-Alert high-density seismic monitoring network during various earthquakes in Taiwan is cited by the NTU researching team, particularly, in terms of EEW, and providing detailed shakemaps to disaster relief agencies for earthquake disaster prevention and risk mitigation [15,22,23]. The damage scenario during an earthquake can be represented using shakemaps. In recent times, to utilize the network efficiently, the network is upgraded to plot the PGV, the CWB Intensity scale, *S_a,_* and even coseismic displacement shakemaps [24], besides, PGA shakemap.

In the regional warning process, the warning time depends on the distance of the recording station from the epicenter and the speed of data transmission from the field instrument to the central recording station. The earthquake can be detected promptly if the recording instruments are closer to the epicenter. Also, the accurate warning is a function of more than one instrument, so the detecting network should be denser. A considerable error is reported in the earthquake location when the earthquake occurs either at the border or outside the instrumentation monitoring window. Because of the erroneous location, there may be a delay in estimating the shaking and subsequently issuing the warning to the affected areas. As *P*-Alert instruments are installed densely in every part of Taiwan, the in-land earthquakes are reported accurately with minimum error. The offshore events on the other hand report a little higher error in location but still are manageable [25]. The data transfer in the regional warning is crucial as delay in packaging and sending the signal to the central computing server may cause a delay in parameter calculation and eventually the issuing of warning [26]. To ensure that the data transfer may not cause an additional delay in issuing warnings, all the instruments are mainly installed in elementary schools, and each instrument is assigned a dedicated stable ethernet connection. The significance of the MEMS seismic instruments for seismic monitoring has been established in the past few years. In recent times, some countries are working for EEW using low-cost sensors. As *P*-Alert instruments are cost-effective, the *P*-Alert network is updated every month to include additional 5-10 instruments. The distribution of all P-Alert instruments installed in Taiwan is shown in Figure 1.

## 3. Shakemaps

In early times, the earthquake magnitude and location were the two early warning parameters available after the occurrence of an earthquake. Later on, it was realized that additional details are required to access the damage pattern. An earthquake that has one magnitude and location will depict different shaking scenarios corresponding to different parameters like PGA and Intensity. The property damage can be reduced if the emergency services are directed to earthquake-hit areas at the earliest. So, this information related to affected areas can be gathered from shakemaps, such as Intensity shakemap, PGA shakemap, etc. A shakemap can demonstrate the ground shaking at various locations of a region. The conventional way of generating a shakemap is the combination of ground-motion prediction equations (GMPEs), EEW parameters, site corrections, and partial observation values [27]. However, due to the limitation of the point source from applying GMPEs, those near-source and finite fault effects are barely seen on this kind of shakemaps. It is hard to do a perfect finite rupture model to approximate the real scenario just like that we can’t handle the real fault rupture as well. Still, there are other ways to achieve the goal of presenting details based on different parameters other than magnitude and location. There is an uncomplicated method that is simply based on increasing the amount of the seismographs to achieve a denser and more uniform spacing earthquake monitoring network. With this kind of dense network, we can directly integrate the measurements from each instrument and do the interpolation to derive a highly detailed shakemap with near-source and finite fault information. This kind of measurement would be a really difficult challenge with traditional sensors but is achievable using the NTU P-Alert network that is all consisted of MEMS sensors, *P*-Alerts.

The *P*-Alert network till 2019 has published near real-time PGA shakemaps during and after the occurrence of an earthquake. In general, P-Alert starts plotting shakemaps once 10–12 instruments confirm PGA to be 0.012g. These maps provide an idea about the rupture directivity, as well as radiation patterns [23,26]. Initially, the NTU network was used to plot PGA shakemaps only. However, later on, it was realized that only PGA maps were not enough to carry out additional studies. Sometimes, PGA maps are not sufficient to locate the earthquake-ravaged areas and represent the damage pattern effectively and so the information provided to the earthquake disaster management authorities may be inadequate. In recent times, two earthquakes having the same magnitude that occurred in 2018 and 2019 in Hualien county of Taiwan produced quite different damage scenarios. The PGA shakemaps were the same for both earthquakes in the epicentral region. It was only the PGV shakemap that depicted that the earthquake having higher PGV values caused more destruction. Some other studies hinted that the PGV based shakemaps are more indicative of the actual disaster area [28] than the distribution of the PGA. Therefore, to make the maximum utilization of this network in the field of earthquake disaster prevention and reduction, we have upgraded the present system to plot the additional shakemaps. These additional shakemaps will benefit significantly the end-users. Right now after the upgradation, the P-Alert system in addition to PGA shakemaps can provide the PGV, *S_a_* at different periods, and the CWB Intensity scale shakemaps. The CWB Intensity scale represents the damage patterns or felt intensity by the human. Also, *S_a_* maps at different periods (0.1 s, 0.3 s, and 1.0 s), corresponding to different story buildings are plotted. *S_a_* represents the maximum force experienced by the buildings having a particular natural vibration period.

This *P*-Alert system also publishes the earthquake information and the resulting shakemaps to social media within a few seconds after the earthquake origin. We believe that with upgradation in the present network, such real-time and needed information is easily available, and with the help of this information, the disaster relief resources can be delivered to disaster-hit areas in a fast and appropriate way.

## 4. 2018 and 2019 Earthquakes

In the eastern part of Taiwan, near Hualien County, two earthquakes having almost the same magnitude occurred in 2018 and 2019. The CWB rapid-reporting system located the 2018 earthquake 18 km northeast of the Hualien city having M_L_ 6.2. This earthquake caused the highest fatalities in recent times, most of them from a multi-storied hotel building. The 0.6g PGA corresponding to the CWB Intensity VII was recorded at one or two stations during this earthquake. The PGA shakemaps during the earthquake were available within 2 min of the occurrence of the earthquake. Another Hualien earthquake having M_L_ 6.3 occurred in 2019, exactly 14 months later of the 2018 earthquake. The CWB rapid-reporting system, located this earthquake 10 km northwest of Hualien County with a focal depth of 18.8 km. The recorded PGA during this earthquake reached 0.5 g.

**Figure 2:**
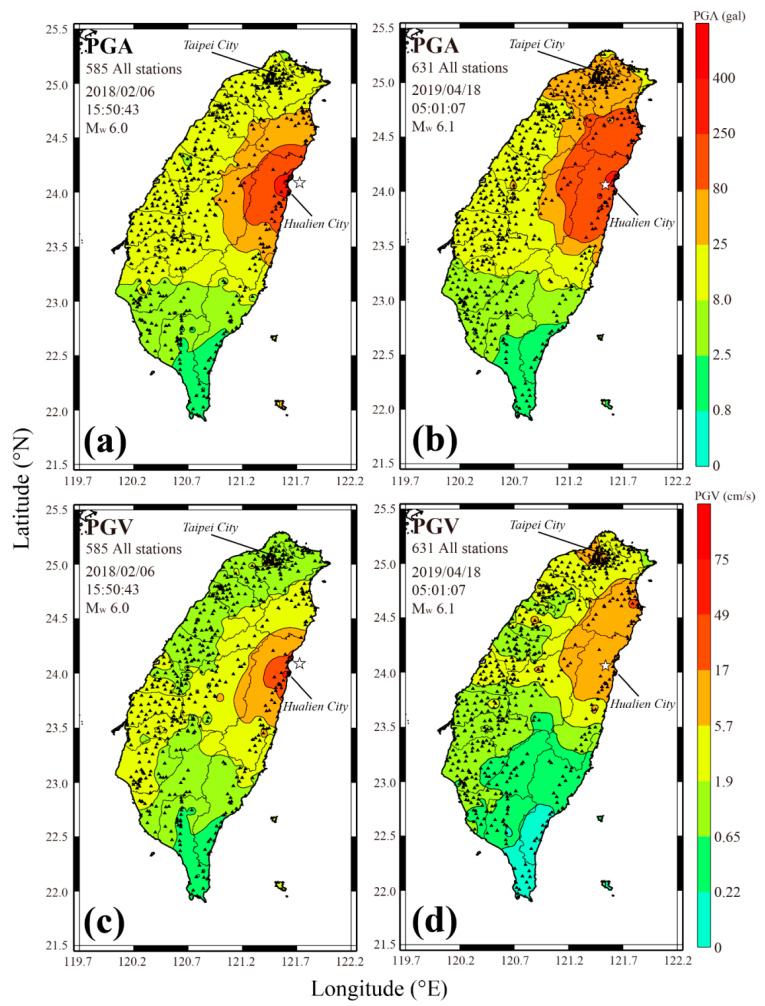
The distribution of PGA (**a**,**b**) and PGV (**c**,**d**) during the 2018 and 2019 Hualien earthquakes. Although, higher PGA values are obtained during both the earthquakes, the damage is concentrated in an area of high PGV.

The higher PGA values (˃ 0.4 g) were observed at a few stations in the epicentral region during the 2018 earthquake. The higher PGV values (PGV ≥ 75 cm/s) are expected in the epicentral region corresponding to higher PGA values (PGA ≥ 0.4 g). However, looking at the PGV contour map for this earthquake (Figure 2), a small contour of PGV ≥ 49 cm/s is observed, to the southwest of the epicenter where maximum destruction was caused. Looking at the other higher PGA values areas (PGA ≥ 0.25g) and seeing the relation between PGA and PGV values in Taiwan, higher PGV values (PGV ≥ 49 cm/s) are expected in those areas. However, no such phenomenon was observed but the devastation was concentrated in an area having higher PGV values (away from the area of higher PGA). During the 2019 earthquake, the 0.025 g PGA contour spread in a larger area, including Taipei city (capital region). In Taipei city, PGA values between 0.025–0.08 g were observed during this earthquake, which corresponds to 5.7–17.0 cm/s PGV contour. The PGA contour (0.025–0.08 g) spread in a bigger area but the corresponding PGV contour was confined in the Taipei area only. Looking at the PGA values in Taipei city, no destruction is expected. Still, a building in Taipei city leaned against its neighbor after this earthquake, indicating that PGV may be a better indicator of destruction scenario as compared to the PGA.

## 5. The Shakemap Methodology Upgradation

**Figure 3:**
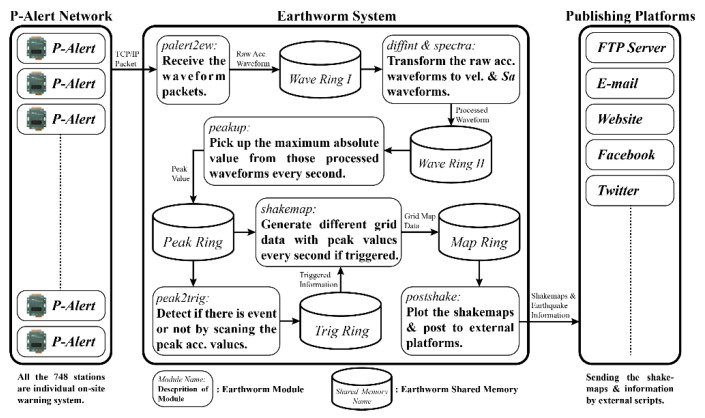
A flowchart showing the different steps and modules involved in plotting the different shakemaps (PGA, PGV, Intensity, and *S_a_*).

The present work related to shakemaps upgradation uses the data collected by the P-Alert network. The research system is based on the Earthworm platform [3,29] that was initially developed by U.S. Geological Survey (USGS), and several other unique modules have been developed and integrated into the original platform. The updated functioning structure of the P-Alert network is described in Figure 3. Following is the detailed introductions to each module.

Firstly, the data receiving part includes only one module, ***palert2ew***. It is used as a server-side program waiting for connections from *P*-Alert sensors across Taiwan and then receives the waveform data packets through TCP/IP protocol. Besides, the module can also be used as a client-side program connecting to the main *P*-Alert server to request the waveform data from all online *P*-Alert sensors. The main process inside this program is parsing the raw *P*-Alert packet, and then transforming it to the Trace Buffer format in the Earthworm platform. Once the data is processed, it is transferred to the first shared memory, ***Wave Ring I***.

For data in ***Wave Ring I***, two different additional actions are performed in the data processing part. In the first action, the data is handled by the differential and integral module, ***diffint***. This module first cooperates with the conversion parameters in the database to convert integer records into real physical quantities (gal) and then using integral, convert the original acceleration waveforms into velocity and displacement waveforms. To avoid the noise-induced drifting, we also apply the high pass filter that is two poles Butterworth filter at 0.075 Hz corner frequency. Finally, output the results to the next shared memory (***Wave Ring II***) in the Earthworm platform. The other action is performed by the calculation module, ***spectra***. This module calculates the corresponding response *S_a_* based on the defined period and damping ratio. This process is more complicated than the previous due to the real-time transformation between acceleration and *S_a_* signal. Conventionally, the transformation is performed using Fourier transform and Inverse Fourier transforms but it takes too much time and computing power. Therefore, we use another method that is similar to the recursive filter [30]. The detailed equation is as follows:

(1) $${\mathrm{Sd}}_{i}=\frac{1}{c_{1}}\left[a_{i}\times {\mathrm{dt}}^{2}+2\times c_{2}\times {\mathrm{Sd}}_{i-1}-{\mathrm{Sd}}_{i-2}\right]$$

(2) $$c_{1}=1+h\times \omega_{0}\times \mathrm{dt}$$

(3) $$c_{2}=1+2h\times \omega_{0}\times \mathrm{dt}+{{(\omega }_{0}\times \mathrm{dt})}^{2}$$

(4) $$\omega_{0}=2\pi /T$$

The ${\mathrm{Sd}}_{i}$ is the spectral displacement of the ith sample, the $a_{i}$ is the acceleration record of the ith sample, the dt is the delta time interval between two samples. The *h* is the damping ratio that is set to 0.05 as usual. After this recursive processing, we will derive the spectral displacement record. After applying another double differential, the *S_a_* is obtained. The output from this module is also transferred to the ***Wave Ring II***. Next, the scanning module, ***peakup***, scans all of the waveforms including acceleration, velocity, displacement, and *S_a_* inside the ***Wave Ring II*** once per second, corrects the zero offsets, finds the maximum value of the waveform per second, and finally, outputs the results to the next shared memory (***Peak Ring***).

Following is the trigger process part which includes only one module, ***peak2trig***. This module will collect the peak value information generated by the previous module to determine whether a possible earthquake event has occurred. Once the triggering stations reach more than five, it is defined as an earthquake event. The optimum condition for the triggering station is that the maximum acceleration reaches 0.0015 g in one second, and the other two stations within 30 km also report 0.0015 g within 6 s. Once triggered, this module will continue to update the trigger information and output the trigger information to the shared memory (***Trig Ring***). A simple linked list data structure is used inside this module to implement this algorithm. In this way, we can manage the triggered station list easily such as removing the obsolete triggered station or attaching the incoming triggered station. To this portion, all the data for generating shakemap are ready.

**Figure 4:**
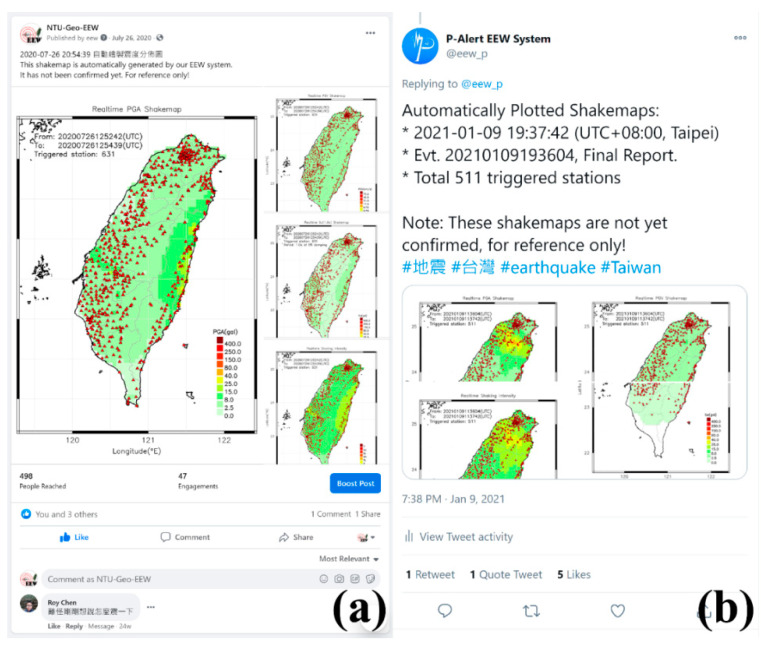
The information posted on social media like Facebook **(a)** and Twitter **(b)** within seconds after the earthquake occurrence.

Then the mapping process part has two main modules. The first module is ***shakemap***, it will receive the peak value information from the ***Peak Ring*** continuously and simultaneously detect whether there is trigger information from ***Trig Ring***. Once it is triggered, the ***shakemap*** module will enter the triggered mode and save the peak value of each station. Also, it will perform the spatial interpolation calculation using inverse distance weighting [15,31,32,33,34] once per second to obtain a reasonable regional shakemap. In addition to sending the calculation result to the shared memory (***Map Ring***), it also sends the output to the local hard disk in a text format for archiving. The second module is the drawing and publishing module, ***postshake*.** It firstly draws the image file (png format) of regional shakemap according to the data generated by the previous module; once all required shakemaps are drawn, this module will send the emails with earthquake message notifications to the address on the list. Also, the message is posted to social media, such as Facebook, Twitter (started posting on Twitter in October 2020) by calling external scripts (Figure 4).

## 6. A Real Example of The 26 July 2020 Earthquake

The upgraded P-Alert system was officially launched in early 2020. Since its operation, only a few earthquakes with a magnitude greater than 5.5 have occurred and were successfully recorded by this network. The functioning of this network is described in terms of one such earthquake having a magnitude M_L_ 6.2 that occurred on 26 July 2020. During the testing period at the end of 2019, many earthquakes were recorded; however, they were included in the testing phase as the system was not officially launched. Like most of the high magnitude earthquakes in Taiwan, the 26 July 2020 earthquake was located offshore in the eastern part of Taiwan. As per the CWB rapid-reporting system, the earthquake having M_L_ 6.2 and depth 53.6 km was located 85 km southeast of Ilan County. It is one of the few highest magnitude earthquakes that occurred in the periphery of Taiwan after the upgradation of the P-Alert network. Although this earthquake did not cause any noticeable destruction in Taiwan, the P-Alert network initiated and plotted different shakemaps. The on-site warning by the P-Alert network is initialized once the predefined thresholds set for warning are exceeded (i.e., *P_d_* ≥ 0.35 cm_,_ PGA ≥ 0.08g). *P_d_* calculation from instruments is a two-staged process that is followed continuously. A 0.08g PGA value in Taiwan equals seismic Intensity V as per the old Intensity scale and IV according to the new scale. These values of PGA, PGV, and Intensity are supposed to cause light damage in the epicentral region [35].

**Figure 5:**
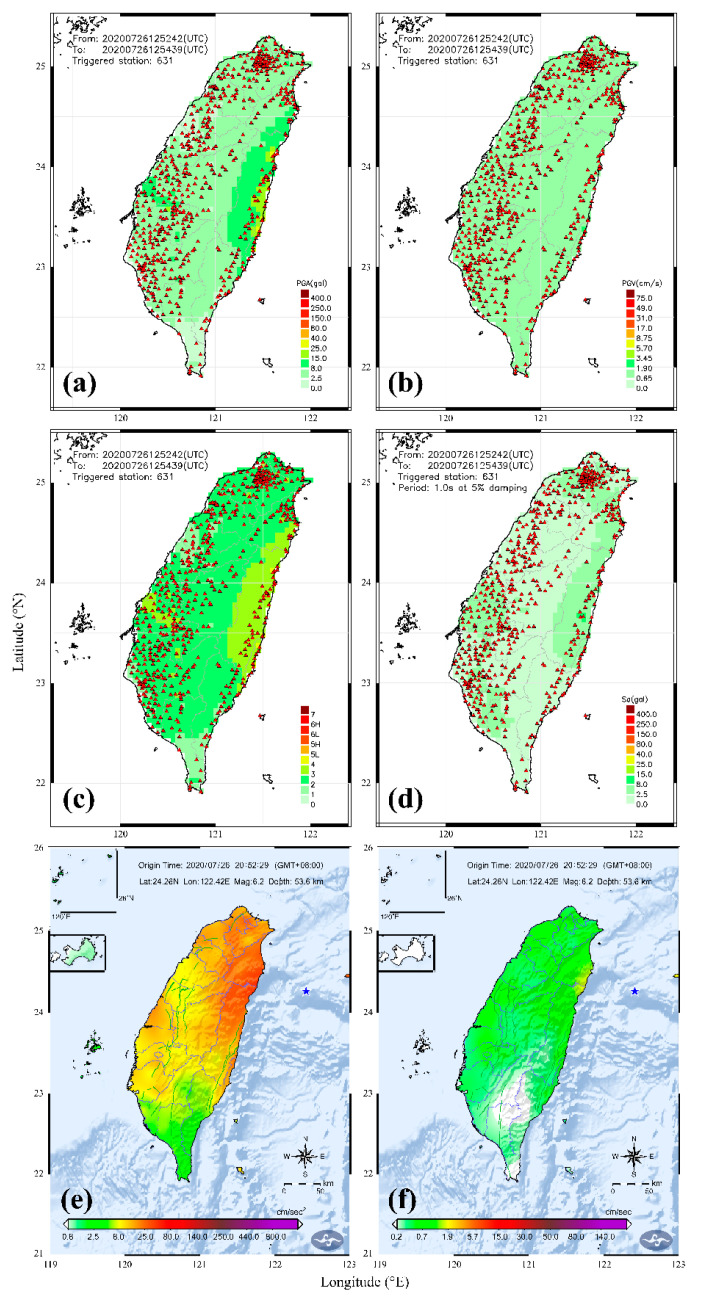
The real-time PGA (**a**), PGV (**b**), Intensity (**c**), and *S_a_* (**d**) maps available within approximately 2 min of the occurrence of the earthquake. The earthquake started at 12:52:29 and the final shakemaps were available at 12:54:39 with a total triggering of 631 stations. The PGA (**e**) and PGV (**f**) shakemaps were posted by CWB at 12:57:52.

**Figure 6:**
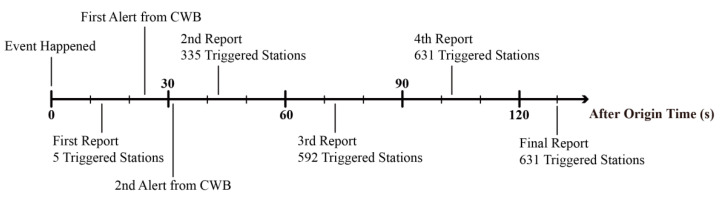
The difference in reporting by *P*-Alert and CWB network. The first report is generated by the *P*-Alert network with the triggering of 5 stations after 13 s of earthquake occurrence. CWB issued the first alert after 24 s and the second alert after 32 s. *P*-Alert generated the 2nd report after 43 s and the final shakemaps with triggering of 631 instruments were available approximately after 130 s.

The earthquake was deep and very far from the Taiwan boundary and the nearest station of the P-Alert network is about 60 km far from the epicenter. By the time, the P-Alert network recorded this earthquake, the PGA values had already attenuated. The PGA and *P_d_* values recorded by instruments were lower than the predefined thresholds, so the EEW system was not initialized. The earthquake started at 12:52:29 (UTC) and the first shakemaps were generated at 12:52:42 just 13 s after the origin time. Then every 30 s, the P-Alert system generated a series of shakemaps continuously. The stable shakemaps were first generated at 12:54:12, and then the final shakemaps were available at 12:54:39 with a total triggering of 631 stations (Figure 5). Comparing to the official report from CWB posted at 12:57:52, we could recognize the damaged area from the detailed shakemaps much earlier. The P-Alert network shakemaps are in agreement with CWB shakemaps, emphasizing that the updated network works perfectly. However, the P-Alert network is quick enough in plotting the real-time shakemaps as compared to the CWB network. The P-Alert network generated the first report with the triggering of 5 stations after 13 s of earthquake occurrence (Figure 6). CWB issued the first alert after 24 s and the second alert after 32s . P-Alert generated the 2nd report after 43 s and the final shakemaps with triggering of 631 instruments were available approximately after 130 s.

## 7. Discussion and Conclusions

The NTU research team started establishing the NTU network in 2010 when the first *P*-Alert instrument was installed in the Hualien region of Taiwan. Since then, the team increased the number of stations by around 50–100 every year and extended the network to cover most of the Taiwan area. Finally, in 2020, it became a network that consists of over 700 stations with a spacing of around 5 km. Among the similar MEMS-based networks, the NTU network is unique due to the selection of installation environments. Deploying *P*-Alert instruments in elementary schools and educational institutes in Taiwan with proper logistics including a dedicated network provides an opportunity to densely instrument the whole Taiwan country and continuously transfer high-quality data to the processing center. Generally, the instruments are placed on the first (about 77%) or second floors (about 19%) of the buildings. However, most of the *P*-Alert instruments are mounted vertically on the walls, so the measurements may be affected by the soil-building interaction. To compute the difference in measurements, Wang et al. [36] collected the data from Taiwan Strong Motion Instrumentation Program (TSMIP) instruments placed close to *P*-Alerts and found the difference in values by dividing PGA values recorded by both of them. They found that the PGA values recorded by the *P*-Alert instruments placed on the ground floor were the same as TSMIP instruments and for instruments at the first and second floor they were 1.07 and 1.52 times, respectively. Therefore, the amplified seismic waveforms from *P*-Alert instruments installed on a wall of different floors can be adjusted directly using the factor mentioned above. After removing the building amplifying effect, the hesitation of the overestimation in shakemaps generated by *P*-Alert is also eliminated.

In Figure 6, it shows that the NTU network can provide the shakemaps report in a really short time. The first report came out even faster than the official alert issued by the CWB. However, in the first report, we can only tell a small area with initial low shaking which might not be useful for early response, and the next report came out after 30 s. This time interval is adjustable in the system setting. The interval can be reduced to even once per second. Avoiding over messages on social media, the 30 s interval is adequate. But, if there are clients who care about the high timeliness, the publish interval can be adjusted separately. And with such high timeliness, these shakemaps will be able to provide some other valuable information like rupture directivity of fault [23,26] in addition to the shaking distribution.

Although the NTU network already becomes a robust and dense network with the *P*-Alert instruments, there are still some upgrades that need to be done. To provide more high-quality waveform data and improve the sensitivity. The advanced *P*-Alert plus instruments which belong to Class-B type [9] will be deployed increasingly among the NTU network in the next few years. Then, the detection ability and data quality of this network will be much better with the 24-bit data providing.

The *P*-Alert instrumentation has been proven to be very effective in generating the various shakemaps and immediately issuing an earthquake early warning to the various lifeline structures including schools. After the upgradation, the *P*-Alert system can provide the PGV, *S_a_* of various periods, and the CWB Intensity scale shakemaps in addition to PGA shakemaps. These additional shakemaps will benefit significantly the end-users which are mainly the disaster management authorities and administrative planners of the country. The upgradation of the network to generate additional shakemaps will be helpful to delineate the earthquake-hit areas and to provide help at the earliest to mitigate the earthquake disaster. The addition of the new instruments regularly and instrumenting on the known faults ensures that the near-fault properties are captured very precisely on this network. This leads to this instrumentation always to be ahead in generating various value-added products to be circulated on social media and delivering them to the disaster-relief agencies at the earliest using a strong communication system.

The next step is to expand this network on the eastern side of Taiwan where the mountainous area and the scarcity of the proper logistics pose difficulty in expanding the network. Though a few instruments have already been added, still, there is room for many other instruments. In the future, we plan to expand the network and to combine the resultant shakemaps with several building components related to construction and design to further generate complete seismic hazard maps for important cities in Taiwan.

## Data Availability

The strong motion waveform records from the P-Alert network used in this study can be downloaded at http://palert.earth.sinica.edu.tw/db/.
